# A Stretchable Scaffold with Electrochemical Sensing for 3D Culture, Mechanical Loading, and Real‐Time Monitoring of Cells

**DOI:** 10.1002/advs.202003738

**Published:** 2021-05-27

**Authors:** Yu Qin, Xue‐Bo Hu, Wen‐Ting Fan, Jing Yan, Shi‐Bo Cheng, Yan‐Ling Liu, Wei‐Hua Huang

**Affiliations:** ^1^ College of Chemistry and Molecular Sciences Wuhan University Wuhan 430072 China; ^2^ College of Chemistry and Chemical Engineering Institute for Conservation and Utilization of Agro‐Bioresources in Dabie Mountains Xinyang Normal University Xinyang 464000 China

**Keywords:** 3D cell culture, chondrocytes, electrochemical detection, mechanotransduction, stretchable scaffolds

## Abstract

In the field of three‐dimensional (3D) cell culture and tissue engineering, great advance focusing on functionalized materials and desirable culture systems has been made to mimic the natural environment of cells in vivo. Mechanical loading is one of the critical factors that affect cell/tissue behaviors and metabolic activities, but the reported models or detection methods offer little direct and real‐time information about mechanically induced cell responses. Herein, for the first time, a stretchable and multifunctional platform integrating 3D cell culture, mechanical loading, and electrochemical sensing is developed by immobilization of biomimetic peptide linked gold nanotubes on porous and elastic polydimethylsiloxane. The 3D scaffold demonstrates very good compatibility, excellent stretchability, and stable electrochemical sensing performance. This allows mimicking the articular cartilage and investigating its mechanotransduction by 3D culture, mechanical stretching of chondrocytes, and synchronously real‐time monitoring of stretch‐induced signaling molecules. The results disclose a previously unclear mechanotransduction pathway in chondrocytes that mechanical loading can rapidly activate nitric oxide signaling within seconds. This indicates the promising potential of the stretchable 3D sensing in exploring the mechanotransduction in 3D cellular systems and engineered tissues.

## Graphical abstract

In this work, a stretchable three‐dimensional (3D) scaffold is developed with peptide linked gold nanotubes on porous and elastic polydimethylsiloxane, which integrates with functions of cell culture, mechanical stimulation, and powerful detection capability for the first time. The proposed scaffold is used for real‐time monitoring of stretch‐induced nitric oxide release from chondrocytes in exploring the mechanotransduction in 3D cellular systems.

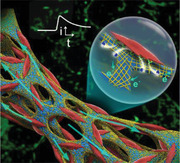

## 1 Introduction

Cells in vivo reside in a three‐dimensional (3D) dynamic microenvironment consisting of soluble factors, cell‐matrix interactions, cell–cell contacts, and specific physicochemical properties.^[^ ^1^ ^]^ Over the past decades, to better mimic such physiological conditions in vivo, 3D cell culture systems have emerged and greatly boosted the development of many fields such as tissue regeneration, pathophysiological study, and drug screening.^[^ ^2^ ^]^ Among them, scaffolds‐based 3D cell culture for cell adhesion, proliferation, and extracellular matrix production has attracted extensive attention,^[^ ^3^ ^]^ due to the effective nutrient transport, similar structures to native extracellular matrix, and tunable mechanical properties.^[^ ^1b^ ^]^ Therefore, research focus has long centered on 3D scaffolds fabrication based on various materials (e.g., hydrogels of naturally derived protein or proteoglycan, synthetic polymers, and composite materials) and techniques (e.g., electrospinning and 3D printing).^[^ ^2^, ^4^ ^]^

For 3D cell culture, besides the “closer‐to‐in vivo” architectures and physical cues provided by scaffolds, mechanical loading has attracted increasing attention in recent years as its critical role on cell functions such as migration, growth, differentiation, and survival.^[^ ^1a^ ^]^ Mechanical loads are especially representative for resident cells in tissues such as ligament, bone, heart, muscle and so on,^[^ ^5^ ^]^ and these cells can sense and translate mechanical signals into biochemical responses via sophisticated mechanotransduction process.^[^ ^6^ ^]^ In this regard, 3D scaffolds (e.g., collagen hydrogels,^[^ ^7^ ^]^ fibrin matrix,^[^ ^8^ ^]^ and polyurethane porous scaffold^[^ ^9^ ^]^) with deformable properties were used as the support to culture and investigate these cells under mechanical loading including tensile strain,^[^ ^7^ ^]^ shear stress,^[^ ^8^ ^]^ and compression.^[^ ^9^ ^]^ Generally, the cellular responses to mechanical loading were investigated by characterizing cell morphology, marker proteins and gene levels, and such evidences were reported by the immunostaining, western blot, and polymerase chain reaction, after cells were subjected to mechanical stimulation for several hours or even days.^[^ ^10^ ^]^

Nevertheless, what appears to be missing in the exciting advances is techniques for real‐time acquiring the dynamic information during cell mechanotransduction, since mechanical signals could trigger rapid biochemical responses within a second.^[^ ^11^ ^]^ To solve this problem, it will be a convincingly preferred approach to develop 3D scaffolds that could integrate with functions of cell culture, mechanical stimulation, and real‐time detection capability,^[^ ^12^ ^]^ but no such a scaffold has been reported so far. Electrochemical sensing is a well‐accepted forceful technique capable of real‐time monitoring of chemical molecules from living cells or tissues with fast response and excellent sensitivity.^[^ ^13^ ^]^ Recently, stretchable electrochemical sensors have emerged and demonstrated an enormous advantage for real‐time inducing and monitoring of cell mechanotransduction with cells cultured thereon.^[^ ^14^ ^]^ However, the reported stretchable sensors work in 2D planar form, and the very few 3D scaffolds based on graphene and conducting polymer^[^ ^15^ ^]^ have great difficulty in withstanding large mechanical deformation for lack of elasticity. So far, no breakthrough has been made in fabrication of stretchable 3D electrochemical sensors for inducing cell mechanotransduction while investigating it in real‐time.

**Scheme 1:**
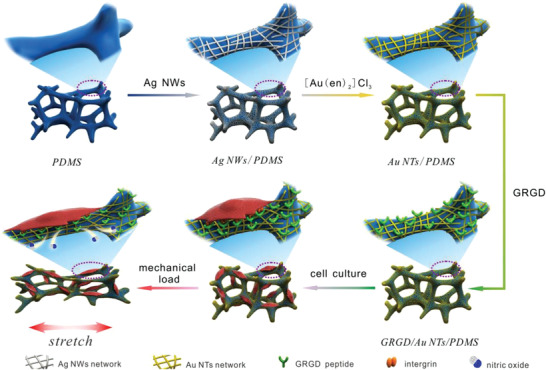
Schematic of GRGD/Au NTs/PDMS scaffold fabrication and the monitoring of cell cultured thereon upon application of a stretching load.

Aiming at this, we developed a stretchable and biomimetic 3D scaffold with electrochemical sensing performance, which was fabricated by attaching the networks of peptide linked gold nanotubes (Au NTs) on porous polydimethylsiloxane (PDMS). Herein, an elastic 3D PDMS scaffold was first replicated from porous Ni foam, and uniform Au NTs networks thereon were obtained via galvanic displacement with silver nanowires (Ag NWs) as sacrificial template. To improve cell attachment on the scaffold, Gly‐Arg‐Gly‐Asp (GRGD) peptide was linked with Au NTs via Au—S covalent bond. As expected, the prepared GRGD/Au NTs/PDMS scaffold possessed very good compatibility to host cells thereon, reproducible mechanical deformation while retaining stable and sensitive electrochemical response. As a proof of concept, the multifunctional scaffold was used as a chondrocytes culture platform to mimic 3D structure of cartilage and was then subjected to different strains to load mechanical stimuli. Using this unique scaffold, the stretch‐induced production of signaling molecule, nitric oxide (NO) from chondrocytes was successfully studied (**Scheme** **1**).

## 2 Results and Discussion

### 2.1 Preparation and Characterization of GRGD/Au NTs/PDMS Scaffold

To start with, a flexible PDMS scaffold was replicated from Ni foam,^[^ ^15^, ^16^ ^]^ and then used as the substrate for immobilization of Au NTs by in situ galvanic displacement of Ag NWs with [Au(en)_2_]Cl_3_ (this procedure is similar to that in our previous work^[^ ^17^ ^]^ and was described in detail in Section 4). Since the obtained Au NTs served as electrode material, their density had a significant impact on the electrochemical performance of stretchable scaffold. The concentration of Ag NWs solution was optimized with the aid of scanning electron microscopic (SEM) and cyclic voltammetric (CV) characterizations. The results showed that the density of Au NTs on the PDMS skeleton increased with the increasing concentration (0.2–5.0 mg mL^−1^) of Ag NWs solutions (Figure S1, Supporting Information). While high concentration (5.0 mg mL^−1^) of Ag NWs resulted in over‐dense Au NTs networks (Figure S1e, Supporting Information) and even formed free‐standing films across the pore, which would block the mass transport between interconnected skeletons of the scaffold (Figure S1f, Supporting Information). Moreover, CVs of different scaffolds in K_3_[Fe(CN)_6_] (a electrochemical probe) solution showed that the redox current at Au NTs derived from 1.0 mg mL^−1^ Ag NWs solution reached the highest value (Figure S2, Supporting Information), and this should be attributed to the trade‐off between Au NTs density and mass transport. Under this condition, the electrode surface area was greatly increased while the mass transport inside 3D scaffold was not unimpeded. Therefore, 1.0 mg mL^−1^ Ag NWs solution was adopted for the fabrication of Au NTs/PDMS scaffold.

**Figure 1:**
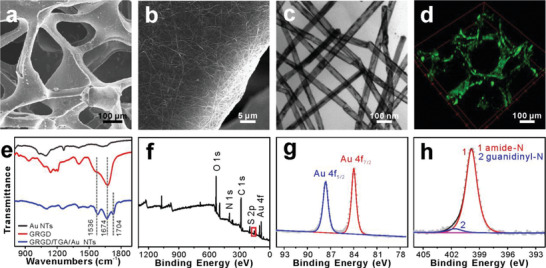
Fabrication and characterization of GRGD/Au NTs/PDMS scaffold. a) SEM image of GRGD/Au NTs/PDMS scaffold. b) High‐magnification SEM image of scaffold surface. c) TEM image of GRGD/Au NTs. d) Laser scanning confocal microscopy image of GRGDK‐FITC/Au NTs/PDMS scaffold. e) FT‐IR spectra of Au NTs, GRGD, and GRGD/TGA/Au NTs. f) The full XPS spectrum of GRGD/TGA/Au NTs. Corresponding spectra of g) Au 4f states and h) N 1s states.

Although Au NTs networks on planar electrodes demonstrated good biocompatibility,^[^ ^17^ ^]^ it was very difficult to seed cells on the 3D Au NTs/PDMS scaffold due to detachment of cells from the scaffold by gravity. In order to enhance cell adhesion, thioglycolic acid (TGA) monolayer was self‐assembled onto the surface of Au NTs via Au—S bond, and the —COOH groups were used to covalently linked with a cell adhesion promoting peptide GRGD by amide bond, forming the biomimetic 3D GRGD/Au NTs/PDMS scaffold. SEM images (**Figure** **1**a,b) showed that the introduction of GRGD had no effect on the morphologies of both 3D PDMS scaffold and Au NTs networks. Besides, transmission electron microscopy (TEM) image (Figure 1c) revealed that the tubular Au NTs still possessed the smooth surface after GRGD modification, which was beneficial for the conductive and electrochemical stability under mechanical strains. Furthermore, FITC‐labeled Gly‐Arg‐Gly‐Asp‐Lys (GRGDK) peptide was employed and treated with the same procedure as that of GRGD, and the clearly observed fluorescent scaffold (Figure 1d) demonstrated the successful immobilization of GRGD.

Fourier transform infrared (FT‐IR), X‐ray photoelectron spectroscopy (XPS), and energy‐dispersive X‐ray spectroscopy (EDX) characterizations were also conducted to further confirm the modification of GRGD molecules. FT‐IR spectrum of GRGD/TGA/Au NTs showed the characteristic absorption peaks of amide I (1674 cm^−1^) and amide II (1536 cm^−1^) at the same position as that of pure GRGD (Figure 1e).^[^ ^18^ ^]^ The full XPS spectrum of GRGD/TGA/Au NTs displayed the presence of Au, S, C, N, and O elements (Figure 1f–h, Figure S3, Supporting Information), in which Au, S, and N corresponded to Au NTs, TGA, and GRGD, respectively. Specifically, the peaks of Au 4f_7/2_ at 84.0 eV and Au 4f_5/2_ at 87.6 eV in Au 4f spectrum were attributed to Au (0) of Au NTs (Figure 1g),^[^ ^19^ ^]^ and N 1s spectrum proved the presence of amide‐*N* (400.3 eV) and guanidinyl‐*N* (401.8 eV) in GRGD (Figure 1h).^[^ ^20^ ^]^ The SEM‐EDX mapping images demonstrated the uniform distribution of S and N elements on the surface of Au NTs (Figure S4, Supporting Information). All these characterizations indicated that the GRGD peptides had been successfully immobilized on the Au NTs/PDMS scaffold. In addition, the optimal concentration (50 µg mL^−1^) of GRGD solution was determined by electrochemical properties and SEM images of the final scaffolds (Figure S5, Supporting Information).

### 2.2 Mechanical Property and Electrochemical Stability of GRGD/Au NTs/PDMS Scaffold

**Figure 2:**
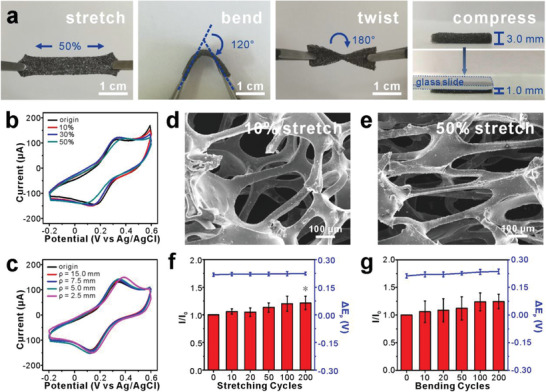
Mechanical behaviors of GRGD/Au NTs/PDMS scaffold. a) Photos showing the flexibility of the GRGD/Au NTs/PDMS scaffold. CVs for 1 × 10^–3^ m K_3_[Fe(CN)_6_] recorded at GRGD/Au NTs/PDMS scaffold with different b) stretches and c) bending radii. SEM images of GRGD/Au NTs/PDMS scaffold with d) 10% and e) 50% stretch. Current intensity (column) and potential difference (line) statistics of CVs for 1 × 10^–3^ m K_3_[Fe(CN)_6_] after different f) stretching and g) bending cycles (data presented as mean ± standard error, *n* = 3, *p*‐values were calculated using one‐way ANOVA, *p < 0.05 vs original state).

Because the scaffold was based on elastic PDMS, the prepared GRGD/Au NTs/PDMS scaffold was capable of bearing various mechanical deformations, including being stretched, bent, twisted, and compressed (**Figure** **2**a). The Young's modulus of the 3D biosensor was also tested and calculated as 33.0 ± 8.7 kPa at 0–10% and 66.2 ± 13.5 kPa from 20% till broken (*n* = 3). The smaller modulus compared to PDMS films (0.6–1.4 MPa)^[^ ^21^ ^]^ might ascribe to that porous structure could disperse and reduce the stress. In addition to the stretchability of the scaffold, it is critical to maintain the initial conductivity and electrochemical properties at the same time for real‐time sensing of mechanically stimulated cells.

To investigate the electrochemical stability, GRGD/Au NTs/PDMS scaffold was mounted on a sliding motor and connected to an electrochemical workstation. When the scaffold was stretched to different stains (10–50%) or bent to different radii (2.5–15.0 mm), CVs in K_3_[Fe(CN)_6_] solution were recorded to evaluate the electrochemical performance. As shown in Figure 2b,c, the peak current and potential of ferricyanide showed high repeatability even when the scaffold was stretched to 50% and bended with a radius of 2.5 mm. It should be explained that the excellent stability lies in two aspects. When the scaffold was under lower strain (10%), the deformation of porous structure was enough to offset the applied stress by reducing the pore size (Figure 2d). As stretch amplitude increased (50%), Au NTs on the skeleton would rotate and slide against each other,^[^ ^22^ ^]^ thereby form continuous electronic pathways (Figure 2e). This stretch strategy brought another benefit that the effective area of Au NTs‐based electrode did not change and mechanical loading had little influence on the current signals (Figure S6, Supporting Information).

Further, the stability of this scaffold was also investigated after being stretched to 10% or bent with a radius of 5.0 mm for 10, 20, 50, 100, and 200 times. The oxidation peak current before and after deformation was specified as *I*_0_ and *I*, respectively, and the potential difference between oxidation and reduction peaks was Δ*E*_p_. The statistics of *I*/*I*_0_ and Δ*E*_p_ were not statistically significant except 10% stretch for 200 times (*p* = 0.04) which indicated that the electrochemical performance was hardly changed after repeated deformations (Figure 2f,g). Taken together, the GRGD/Au NTs/PDMS scaffold had excellent mechanical property while retained electrochemical stability when deformed.

### 2.3 Electrochemical Sensing Performance of GRGD/Au NTs/PDMS Scaffold

**Figure 3:**
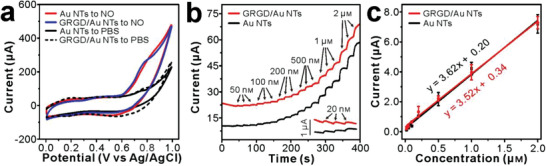
Electrochemical behaviors of GRGD/Au NTs/PDMS scaffold to NO. a) CVs for PBS with and without 100 × 10^–6^ m NO at GRGD/Au NTs/PDMS and Au NTs/PDMS scaffolds. b) Amperometric responses of GRGD/Au NTs/PDMS and Au NTs/PDMS scaffolds poised at +0.8 V versus Ag/AgCl to increasing NO concentrations. c) The calibration curves for the scaffolds in (b) (data presented as mean ± standard error, *n* = 3).

This stretchable and multifunctional scaffold is designed to mimic the cellular microenvironments of 3D tissues and to simultaneously monitor their mechanotransduction process under physiological and pathological conditions in real time. Hyaline cartilage is a connective tissue that covers on the joint and contributes to bone growth and protection, which is always subjected to mechanical strains.^[^ ^23^ ^]^ Osteoarthritis (OA) is a kind of degenerative joint disorder, and it is closely associated with mechanical strains by overproduction of NO,^[^ ^24^ ^]^ which can increase the expression of matrix metalloproteinases, inhibit the matrix protein synthesis, and promote chondrocyte apoptosis and cartilage degradation finally.^[^ ^24a^ ^]^ Considering NO is noted as a potential biomarker of OA, we evaluated the electrochemical response of the scaffold toward NO. Compared with the response of GRGD/Au NTs/PDMS scaffold in PBS, there was an obvious oxidation peak at +0.8 V on the CV curve recorded in 100 µм NO solution (**Figure** **3**a), which indicated that the current change was generated by the oxidation of NO on this stretchable scaffold. Besides, the almost identical responses of Au NTs/PDMS and GRGD/Au NTs/PDMS revealed that the modification of GRGD peptide did not deteriorate the electrochemical sensing performance of original Au NTs.

The amperometric responses of GRGD/Au NTs/PDMS and Au NTs/PDMS to a series of NO concentrations were shown in Figure 3b, and even low concentration of 20 × 10^–9^ m NO could be sensitively detected (Figure 3b, inset). Both scaffolds displayed linear and reproducible responses to the oxidation of NO in the wide concentration range from 20 × 10^–9^ m to 2 × 10^–6^ m (Figure 3c), and the detection limit of GRGD/Au NTs/PDMS was calculated to be about 9 × 10^–9^ m (*S*/*N* = 3). These results showed that the stretchable GRGD/Au NTs/PDMS scaffold had very sensitive and fast response to the electro‐oxidation of NO. Besides, in view of the inherent excellent electrocatalytic performance of Au NTs, the amperometric responses to the drugs used in subsequent cell detection and typical species with electroactivity were also investigated (Figure S7, Supporting Information). Although some electroactive molecules were detectable on the GRGD/Au NTs/PDMS scaffold, the addition of NO resulted in much larger current increase than those of the interferents with the same concentration (500 × 10^–9^ m). Further considering that the chondrocytes in vivo only uptake ascorbic acid (AA), uric acid (UA), or dopamine (DA) from extracellular synovial fluid rather than synthesize or secrete them^[^ ^25^ ^]^ and the intracellular concentration of H_2_O_2_ maintained at a low level (≈1–100 × 10^–9^ m) under normal physiologic conditions,^[^ ^26^ ^]^ the satisfying sensitivity and selectivity laid the foundation for real‐time monitoring of the primary and transient NO release during cell mechanotransduction responses.

### 2.4 Chondrocytes Proliferation on GRGD/Au NTs/PDMS Scaffold

**Figure 4:**
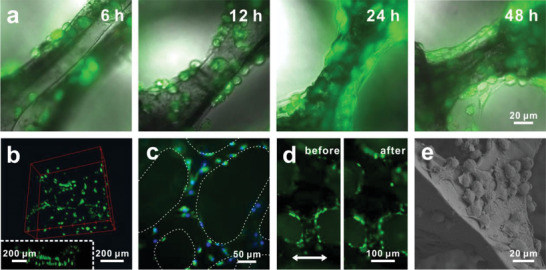
Cell culture and proliferation on GRGD/Au NTs/PDMS scaffold. a) Merged (bright field and fluorescent) images of chondrocytes on GRGD/Au NTs/PDMS scaffold of different culture time and labeled with Calcein‐AM (green) and PI (red). b) Laser scanning confocal microscopy images of 3D construct; inset shows the laser scanning confocal microscopy image on *z*‐plane cross section. c) Immunofluorescent staining of collagen II (green) in chondrocytes on scaffold. Nuclei is labeled with DAPI (blue). d) Fluorescent images of chondrocytes on GRGD/Au NTs/PDMS scaffold before and after 40% stretch. The double arrow demonstrates the stretching direction. e) SEM image for chondrocytes cultured on GRGD/Au NTs/PDMS scaffold for 24 h.

Chondrocytes were harvested from the articulations of 10‐day‐old Sprague–Dawley (SD) rats, and identified by the collagen II immunostaining and toluidine blue staining (Figure S8, Supporting Information).^[^ ^27^ ^]^ Then, the third passage chondrocytes were seeded onto the GRGD/Au NTs/PDMS scaffolds and cultured for different time. As culture time went on, chondrocytes proliferated well on the skeletons and recovered their spindle shapes (**Figure** **4**a), and nearly all cells kept high viability characterized by cell markers 3′,6′‐di(*o*‐acetyl)‐4′,5′‐bis[*N*,*N*‐bis(carboxymethyl)aminomethyl] fluorescein, tetra‐acetoxymethylester (Calcein‐AM, for live cells) and propidium iodide (PI, for dead cells). Confocal microscopic imaging demonstrated that chondrocytes grew all over the surface of the whole 3D scaffold (Figure 4b). Chondrocytes are prone to dedifferentiation during the in vitro monolayer culture,^[^ ^28^ ^]^ and immunostaining of collagen II revealed that chondrocytes cultured on GRGD/Au NTs/PDMS scaffold could maintain their phenotype without obvious dedifferentiation (Figure 4c). However, only a few chondrocytes with low viability and proliferation were observed on the Au NTs/PDMS (Figure S9, Supporting Information) after being seeded and cultured as those on GRGD/Au NTs/PDMS. All these clearly indicated that GRGD peptide immobilized on the Au NTs greatly enhanced cell adhesion and proliferation ability.

Further, the cell adherence was investigated when the scaffold was loaded with 40% stretch. The fluorescent images captured at the same position before and after stretch were almost identical (Figure 4d), and this indicated that chondrocytes could adhere firmly to the surface of the scaffold even under large mechanical loads. SEM further showed that chondrocytes fully outspread on the skeleton surface with numerous pseudopodia at the contact site with the scaffold (Figure 4e). The robust cell adhesion and proliferation laid a good foundation for loading mechanical stretch on chondrocytes via the deformation of GRGD/Au NTs/PDMS scaffold.

### 2.5 Real‐Time Monitoring of Stretch‐Induced NO Release from Chondrocytes Cultured on GRGD/Au NTs/PDMS Scaffold

**Figure 5:**
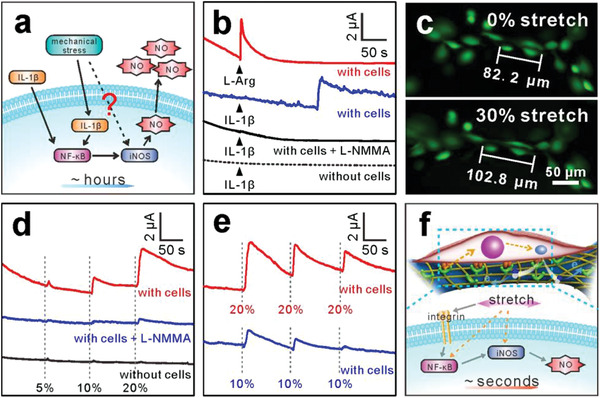
Real‐time monitoring of NO release from chondrocytes cultured on GRGD/Au NTs/PDMS scaffold. a) Schematic diagram representing molecular pathway induced by mechanical stress and IL‐1*β* in long‐term inflammatory reaction. b) Amperometric responses detected from chondrocytes under stimulation of 0.5 × 10^–3^ m L‐Arg or 100 ng mL^−1^ IL‐1*β*. c) Fluorescent images of chondrocytes on GRGD/Au NTs/PDMS scaffold without and with 30% stretch at the same position. d) Amperometric responses detected from chondrocytes under different stretching amplitudes. e) Amperometric responses detected from chondrocytes under 10% and 20% stretches for three times. f) Schematic diagram of the probable cellular mechanotransduction mechanism after mechanical loading within seconds. The vertical gray dashed lines in (d) and (e) indicate the beginning points of mechanical stretches; it took 5 s to achieve every desired strains.

As the unique cell in hyaline cartilage, chondrocytes naturally bear dynamic mechanical loading (e.g., stretch and compression), which is in turn an important regulator of chondrocyte metabolic activity.^[^ ^23^, ^29^ ^]^ Mechanical stress in the normal physiological range can maintain homeostasis balance between anabolic and catabolic activities,^[^ ^30^ ^]^ while excessive mechanical loading is regarded as a crucial factor in the pathogenesis of OA. Excessive mechanical strain induces the production of inflammatory factors (e.g., interleukin‐1 beta, IL‐1*β*) which can further stimulate nuclear factor‐kappa B (NF‐*κ*B) signaling pathway and mediate NO production by regulating inducible nitric oxide synthase (iNOS) expression.^[^ ^31^ ^]^ Most previous studies used indirect methods such as Griess assays^[^ ^32^ ^]^ to measure nitrite ion accumulation after chondrocytes were subjected to mechanical stimulation for several hours or even days. That is, it is still unclear whether mechanical loading could activate NO signaling in the initial stage (**Figure** **5**a).

The ability of the chondrocytes in production of NO was first studied under their unstretched state. 0.5 × 10^–3^ m L‐arginine (L‐Arg), which can be enzymatically oxidated by nitric oxide synthase (NOS) to produce NO, was added to the cell being cultured on the GRGD/Au NTs/PDMS scaffold, and a rapid increase in current was detected (Figure 5b, red line), indicating the normal metabolism activity of chondrocytes. IL‐1*β* is widely recognized as an inflammatory factor in OA models, and an increase in amperometric response was recorded after ≈150 s of IL‐1*β* (100 ng mL^−1^) stimulation (Figure 5b, blue line). Control experiment showed that no obvious amperometric signal could be observed when chondrocytes was pre‐treated with N^G^‐monomethyl‐L‐arginine (L‐NMMA), the total NOS inhibitor (Figure 5b, black line), as well as the case of without chondrocytes on the scaffold (Figure 5b, black dashed line). These results demonstrated that the inflammatory factor IL‐1*β* could actually induce the production of NO via an iNOS‐involved pathway in the chondrocytes. Note that the applied voltage had little influence on the viability and phenotype of chondrocytes, which were confirmed by the Calcein‐AM/PI and collagen II immunostaining (Figure S10, Supporting Information).

The effects of mechanical stretch on NO production by chondrocytes were therefore investigated using the GRGD/Au NTs/PDMS scaffold. With chondrocytes cultured thereon, the scaffold was stretched by a sliding motor to load strains on the chondrocytes. As shown in Figure 5c, chondrocytes could be stretched simultaneously with the scaffold and remain perfectly alive during the stretching process. Then the scaffold and the cells were submitted to 5%, 10%, and 20% stretches to imitate the normal (within 10% strain) and excessive (higher than 10% strain) mechanical loading of chondrocytes.^[^ ^33^ ^]^ It was observed that transient increases in the current were detected within seconds after stretching the cells, and the current spikes increased with the increasing strains (Figure 5d, red curve). 5% strain induced very weak current response, and this is presumably because signaling cascades are force‐dependent and occur only when the force reaches a specific threshold.^[^ ^34^ ^]^ When chondrocytes were pretreated with L‐NMMA, no obvious current change was recorded under the same mechanical loading (Figure 5d, black curve), which indicated that the current increase were caused by NO release from chondrocytes in response to mechanical stretch. The concentrations of generated NO corresponding to 5%, 10%, and 20% stretches were calculated to be 23, 216, and 668 × 10^–9^ m, respectively.

Then, the mechanotransduction responses of chondrocytes subjected to cyclic strains were further investigated. There was a gradual attenuation of the cell responses in both the normal (10%) and excessive (20%) strains (Figure 5e), which was presumably due to a progressive shortage of endogenous L‐Arg for the NO biosynthesis or the feedback regulation by released NO.^[^ ^30b^ ^]^ However, it should be noted that the current spikes caused by 20% strains displayed a much slower attenuation compared with that of 10% strains. It may suggest that chondrocytes could easily adapt to 10% stretch that occurs in normal physiological state, while 20% stretch is excessive mechanical loading for chondrocytes,^[^ ^33^, ^34^ ^]^ which is regarded as the promoter in OA development.

Currently available evidences indicated that NF‐*κ*B pathway is closely related with inflammatory response in OA, in which the upstream IL‐1*β* and downstream iNOS factors were involved.^[^ ^31^, ^35^ ^]^ Given that the inflammatory factor IL‐1*β* requires a certain time (≈150 s as shown in Figure 5b) to trigger NO release, while mechanical stretch‐induced NO production was recorded within seconds. Taken into consideration that integrins act as mechanoreceptors and play an important role in regulating intracellular signaling cascades,^[^ ^36^ ^]^ these results suggest that the mechanical stretch could rapidly activate the NF‐*κ*B signaling or the downstream iNOS existing within the cytosol via force transmission pathway (Figure 5f).

## 3 Conclusion

The recent progress in 3D cell culture calls for effective techniques to acquire the biochemical information in real time, and far more challenging is to quantify transient release of biochemical molecules during cell mechanotransduction process. In this work, we developed a multifunctional 3D scaffold based on GRGD peptide linked Au NTs on porous PDMS, which demonstrated very good compatibility and excellent mechanical performance while retaining stable electrochemical sensing. GRGD peptide modification greatly promoted the adhesion and proliferation of cells on the scaffold, which allowed mimicking the 3D structures of articular cartilage and investigating its mechanotransduction process for the first time. Our results demonstrated that mechanical loading could rapidly activate NO signaling in a force‐dependent manner within seconds. All these indicate that mechanical stretch could rapidly activate the NF‐*κ*B or the downstream iNOS signaling system, possibly disclosing a previously unclear mechanotransduction pathway in chondrocytes.

Yet, the signaling pathways especially the inflammation‐related signaling activated by mechanical loading are very complicated. In the following work, we will devote to the integrated sensor arrays for multiplexed analysis of chondrocyte mechanotransduction. In addition, given the excellent mechanical and electrochemical performance, the combination of our proposed 3D scaffold and hydrogels will be a powerful platform to study many aspects of cell/tissue, such as the effects of various mechanical stress on cell signaling, phenotype expression, tissue remodeling, and so on. Further considering the convenient preparation and excellent mechanical and electrochemical performance, the proposed scaffold combined with rational design is expected to be a versatile platform for 3D cell culture systems to explore the cell/tissue mechanotransduction.

## 4 Experimental Section

### Reagents and Instruments

PDMS liquid prepolymer and cross‐linker were purchased from Momentive Performance Materials (Waterford, NY, U.S.A.). Nickel foam (thickness of 1.5 mm) was bought from Alantum Advanced Technology Materials (Shenyang, China). Dopamine hydrochloride was obtained from Sigma‐Aldrich Corporation (St. Louis, U.S.A.). Ag NWs solution (35–45 nm in diameter, 10–30 µm in length) was purchased from Zhejiang Kechuang Advanced Materials Co., Ltd. (Hangzhou, China). Sodium chloraurate was bought from Aladdin Industrial Co., Ltd. (Shanghai, China). GRGD and GRGDK‐FITC peptides were purchased from Chinese Peptide Company Co., Ltd. (Hefei, China).

Dulbecco's modified Eagle's medium (DMEM) and fetal bovine serum (FBS) for cell culture were bought from GIBCO Corporation (U.S.A.). Collagenase II, bovine serum albumin (BSA), Calcein‐AM, PI, and L‐Arg were supplied by Sigma‐Aldrich Corporation (St. Louis, U.S.A.). Anti‐COL2A1 primary antibody and FITC conjugated goat anti‐rabbit IgG were obtained from Boster Biological Technology Co., Ltd. (Wuhan, China). IL‐1*β* was purchased from GenScript Biotech Corporation (Nanjing, China). Total NOS inhibitor L‐NMMA was bought from Beyotime Biotechnology (Shanghai, China). For all the experiments, deionized water (Millipore, 18.0 MΩ cm) was used. All other chemicals of analytical grade were obtained from Sinopharm Chemical Reagent Co., Ltd. (Shanghai, China) and used as received unless stated otherwise.

SEM images were obtained by field‐emission microscopes (ZEISS SIGMA and Zeiss Merlin compact). TEM (JEM‐2100) was used for characterizing the tubular structure of Au NTs. EDX images were obtained by an INCAPentalFETx3 Oxford EDX spectrometer. XPS measurements were conducted by ESCALAB 250Xi photoelectron spectrometer (Thermo Fisher) and Al K*α* X‐ray radiation was used as the X‐ray excitation source. FT‐IR spectra were measured on a FT‐IR 5700 Spectrometer (NICOLET). Inverted fluorescent microscopes (AxioObserver Z1 and Axiovert 200M, Zeiss) and confocal microscope (PerkinElmer UltraView Vox System, U.S.A.) were used for the observation of scaffolds and cells. Electrochemical measurements were carried out on a CHI 660A electrochemical workstation (CHI Instruments) at room temperature with Ag/AgCl as reference and Pt‐wire as counter electrodes.

### Fabrication of 3D GRGD/Au NTs/PDMS Scaffold

A flexible PDMS scaffold was first obtained by following procedure.^[^ ^15^, ^16^ ^]^ A Ni foam slice (35 × 8 × 1.5 mm^3^, pore size ranges from 40 to 250 µm) was immersed into PDMS prepolymer and centrifuged at 8000 rpm for 5 min. Then the fulfilled foam was transferred into an empty centrifuge tube and centrifuged at 3000 rpm for 3 min to remove excess PDMS prepolymer. The composite was thermally cured at 85 °C for 3 h and the above steps were repeated again. The sacrificial Ni was etched using HNO_3_ solution (7 m), and then the obtained PDMS scaffold was treated with oxygen plasma and immersed in a dopamine solution (1 mg mL^−1^; Tris‐HCl buffer, pH = 8.5) for 24 h for hydrophilization. Subsequently, the scaffold was immersed in an Ag NWs solution (1 mg mL^−1^) to obtain uniformly distributed sacrificial templates. After that, 3D Au NTs/PDMS scaffold was fabricated via in situ displacement of Ag NWs based on previous work.^[^ ^17^ ^]^ The replacement reaction between Ag NWs and [Au(en)_2_]Cl_3_ solution was conducted at 90 °C for 4 h in an oven.

GRGD was linked with Au NTs via Au—S and amide bonds. First, the Au NTs/PDMS scaffold was treated with TGA (50 × 10^–3^ m) in dark for 24 h, and then transferred into 1‐(3‐dimethylaminopropyl)‐3‐ethylcarbodiimide hydrochloride and *N*‐hydroxysuccinimide solution (10 mg mL^−1^ respectively, pH = 4.5) at room temperature to activate carboxyl group for 1 h. At last, the scaffold was reacted with a GRGD peptide solution (50 µg mL^−1^) for 4 h. The final scaffold was washed with PBS solution three times and dried in air.

### Tensile Experiment

Characterization on the Young's modulus of the 3D biosensor was conducted on a universal tensile‐compressive tester (CMT 6503, MTS/SANS, China). The specimens with the width of 80 mm and the thickness of 1.5 mm and the loading rate of 15 mm min^−1^ were used in the tensile experiments. The Young's modulus (*E*) was calculated as

(1) $$E=\left(F\;\times \;L_{0}\right)/\left(A\;\times \;L\right)$$

where *F* is the tensile load, *A* is the cross‐sectional area, *L*_0_ and *L* are the original length and the extended length, respectively.

### Electrode Fabrication and Electrochemical Characterization

3D GRGD/Au NTs/PDMS scaffold was contacted with two tinfoil at both ends via electric paint, and the contact zones were insulated with PDMS prepolymer and then cured at 85 °C to insulate the joints. When used for electrochemical detection, the scaffold was fixed on a PDMS film and precured PDMS was coated around the edge of the scaffold at 85 °C to form a chamber to hold analytical test solution. The final electrode active area was controlled to be 10 × 8 × 1.5 mm^3^ macroscopically. Stretching and bending experiments of 3D GRGD/Au NTs/PDMS scaffold were conducted on a linear sliding motor with a hauling speed of 0.25 mm s^−1^.

### Calibration of GRGD/Au NTs/PDMS Electrode for NO

NO was obtained via disproportionation of NaNO_2_ in H_2_SO_4_ solution and stocked in de‐aerated PBS solution (1.8 mм at 20 °C).^[^ ^17^ ^]^ Calibration for NO was performed by adding a series of NO standard solution aliquots into the stirring PBS solution (5 mL) with GRGD/Au NTs/PDMS electrode immersed in. The electrode potential was held at +0.8 V versus Ag/AgCl for amperometric detection.

### Selective Experiment

The sensor's selectivity was investigated by comparing the amperometric responses for NO to those of the drugs used in cell detection (L‐Arg and L‐NMMA) and typical species with electroactivity (AA, DA, UA, H_2_O_2_, and NO_2_^−^), and the concentration of each component was 500 × 10^–9^ m.

### Articular Chondrocytes Isolation and Culture

SD rats (10‐day‐old, weight of 11–12 g) were purchased from the Hubei Experimental Animal Research Center with the license number SCXK 2020‐0018. Rats were sacrificed by cervical dislocation and cartilage tissue was harvested from the hind limbs as previously described.^[^ ^37^ ^]^ The cartilage was minced and washed three times in PBS (pH = 7.4). The harvested cartilage was treated with trypsin (0.25%) for 45 min to remove fibroblast and terminated by DMEM containing FBS (10%). Then it was digested with collagenase (0.1%) overnight in a humidified incubator to degrade tight extracellular matrix and release chondrocytes. The suspension was filtrated through a sterile cell strainer (pore size of 70 µm) to remove any undigested fragments and the chondrocytes were subsequently resuspended three times with PBS to remove residual collagenase. The cells’ density was determined by a hemocytometer. The collected cells were cultured using DMEM containing FBS (10%), glucose (4500 mg L^−1^), penicillin and streptomycin (100 U mL^−1^, respectively). The third passage chondrocytes were used in optical and electrochemical experiments. To ensure full access to scaffold, trypsinized chondrocytes were seeded and cultured shakily for 30 min before transferring into a humidified incubator.

### Articular Chondrocytes Identification

Chondrocytes were seeded in culture dishes, cultured for 2 days and fixed with paraformaldehyde (4%) for 1 h at 4 °C. For toluidine blue staining, the fixed cells were stained with toluidine blue (1%) for 1 h at room temperature and treated rapidly with ethyl alcohol (95%) as the dehydrating agent. For collagen II immunostaining, the fixed cells were sequentially blocked with 1% BSA for 1 h, incubated with primary antibody (10 µg mL^−1^) overnight at 4 °C and incubated with IgG‐FITC (20 µg mL^−1^) as second antibody and DAPI (1 µg mL^−1^) together for 1 h in a humidified incubator.

### Cell Viability

The viability of chondrocytes cultured on scaffold was assessed by fluorescent live/dead cell markers Calcein‐AM and PI. Cells were washed three times with PBS before cell staining and then Calcein‐AM (2 µg mL^−1^) and PI (3 µg mL^−1^) diluted in PBS were added and maintained for 20 min in a humidified incubator. The stained cells were washed three times with PBS before imaging to reduce fluorescence background.

### Real‐Time Monitoring of NO Release from Chondrocytes

For cell detection, chondrocytes (200 µL, 5 × 10^6^ cells mL^−1^) were seed on sterilized electrode and after being cultured for 24 h to allow cells adhesion, the scaffold was washed three times with PBS to remove culture medium and loosely bonded cells. When monitoring of NO release from chondrocytes, IL‐1*β* (100 ng mL^−1^) and stretching with a fixed stretching time of 5 s were used as stimulation and L‐NMMA (1 × 10^–3^ m) was used as the NOS inhibitor.

### Statistical Analysis

All tests were performed in triplicates. Data were represented as mean ± standard error of the mean. For stability test, the oxidation peak currents were first normalized as *I* /*I*_0_. One‐way ANOVA testing was carried out across groups. In all cases, significance was defined as the probability level *p* ≤ 0.05. Statistical analysis and graph production were performed using software Origin 8.5.

## Conflict of Interest

The authors declare no conflict of interest.

## Supporting information

Supporting Information
