# Supplementary material for: A Stretchable Scaffold with Electrochemical Sensing for 3D Culture, Mechanical Loading, and Real‐Time Monitoring of Cells
Source: Adv Sci (Weinh). 2021 May 27;8(13):2003738. doi: 10.1002/advs.202003738 (PMC8327466; doi:10.1002/advs.202003738)
Supplement: Supplementary file 1 — Supporting Information [file ADVS-8-2003738-s001.pdf]

WILEY-VCH

© 2020 Wiley-VCH GmbH

ADVANCED SCIENCE
Open Access

Supporting Information

for *Adv. Sci.*, DOI: 10.1002/advs.202003738

Supporting Information

A Stretchable Scaffold with Electrochemical Sensing for 3D Culture, Mechanical Loading and Real-time Monitoring of Cells

Yu Qin, Xue-Bo Hu, Wen-Ting Fan, Jing Yan, Shi-Bo Cheng, Yan-Ling Liu and Wei-Hua Huang**

Supporting Information

A Stretchable Scaffold with Electrochemical Sensing for 3D Culture, Mechanical Loading and Real-time Monitoring of Cells

Yu Qin, Xue-Bo Hu, Wen-Ting Fan, Jing Yan, Shi-Bo Cheng, Yan-Ling Liu and Wei-Hua Huang**

Supplementary Figures

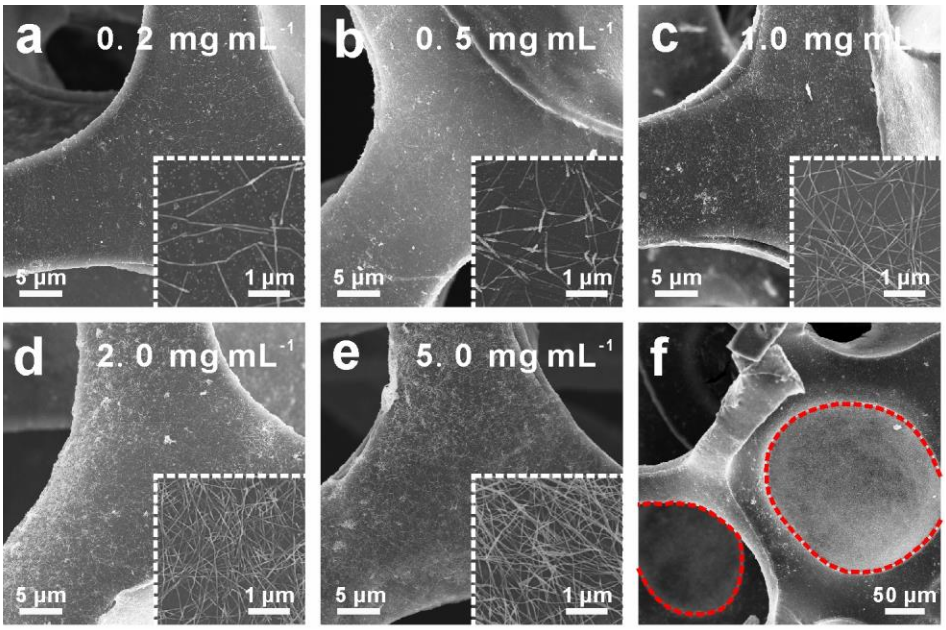

Figure S1. (a-e) SEM images of Au NTs/PDMS scaffolds formed from different concentrations of Ag NWs solution. The insets show corresponding high-magnification images of Au NTs networks on scaffold surfaces (concentrations: (a) 0.2, (b) 0.5, (c) 1.0, (d) 2.0, (e) 5.0 mg mL^{-1}, respectively). (f) SEM image of Au NTs/PDMS scaffold formed from 5 mg mL^{-1} Ag NWs solution. The areas surrounded by red dashed lines demonstrate free-standing Au NTs networks.

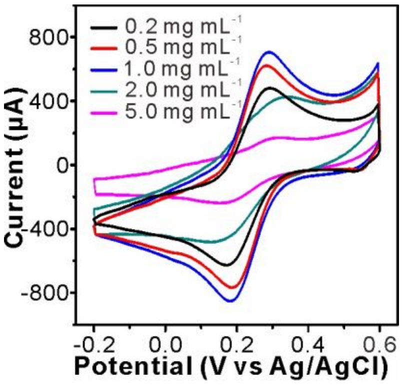

Figure S2. CVs for 1 mM $K_3[Fe(CN)_6]$ recorded at Au NTs/PDMS scaffolds displaced from Ag NWs solution of different concentrations.

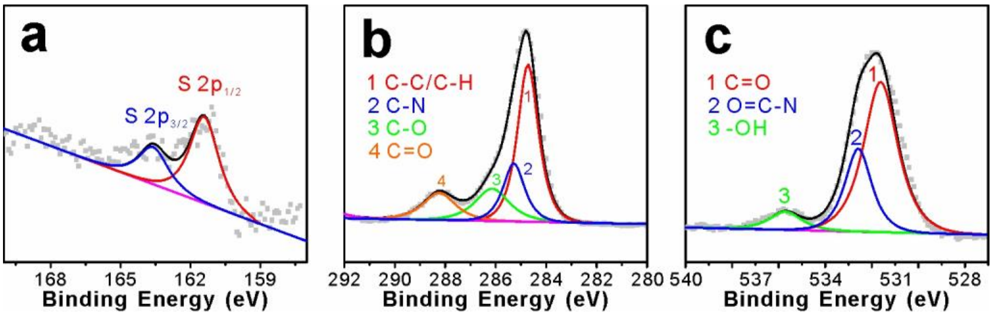

Figure S3. The XPS spectra of S 2p states, C 1s states and O 1s states. (a) S 2p spectrum showed that the peaks of S $2p_{1/2}$ at 161.6 eV and S $2p_{3/2}$ at 163.4 eV were attributed to gold-bound thiolates.[1] (b) C 1s spectrum clearly clarified four typical separated peak components, corresponding to C-H/C-C (284.6 eV), C-N (285.3 eV), C-O (286.1 eV), and C=O (288.2 eV) groups in GRGD/TGA/Au NTs, respectively.[1a,2] (c) The peaks of O 1s spectrum at 531.5, 532.6 and 535.7 eV were due to the C=O bond, O=C-N bond and -OH bond in GRGD, respectively.[3]

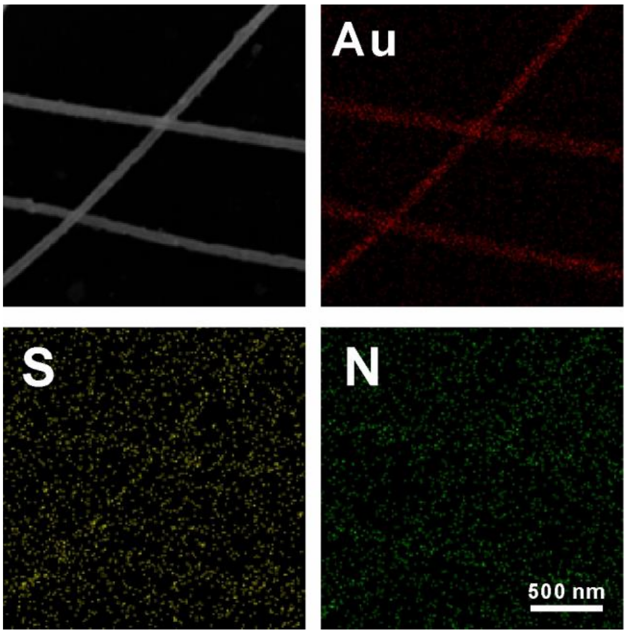

Figure S4. SEM image of GRGD/Au NTs and corresponding EDX elemental mapping images of Au (red), S (yellow) and N (green).

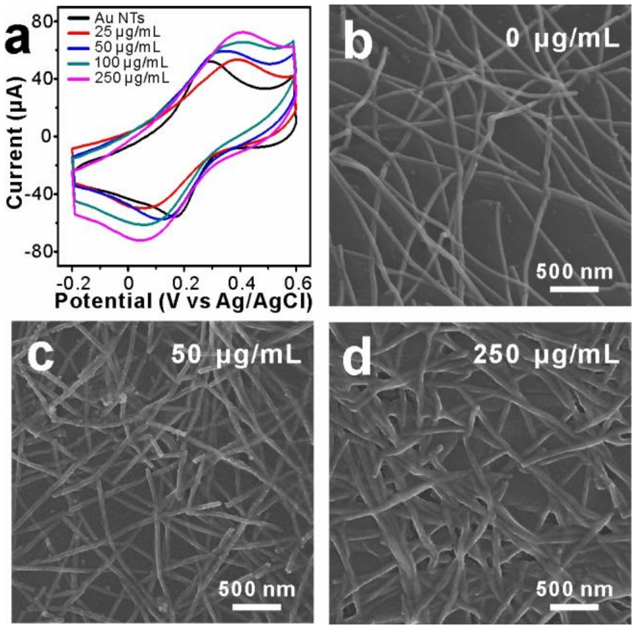

Figure S5. (a) CVs for 1 mм $K_3[Fe(CN)_6]$ recorded at GRGD/Au NTs/PDMS scaffolds modified with different concentrations of GRGD peptide (0, 25, 50, 100 and 250 μg mL^{-1}, respectively) on parallel Au NTs/PDMS scaffolds. (b-d) SEM images of GRGD/Au NTs/PDMS scaffolds modified with different concentrations of GRGD peptide (0, 50 and 250 μg mL^{-1}, respectively).

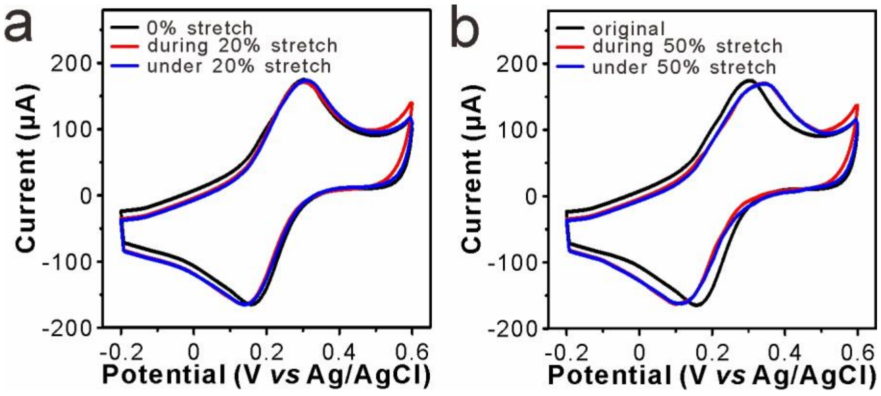

Figure S6. CVs for 1 mм $K_3[Fe(CN)_6]$ recorded at GRGD/Au NTs/PDMS scaffolds without stretch (black line), during cyclic stretch (red line) and under stretch (blue line).(magnitude: (a) 20%, (b) 50%)

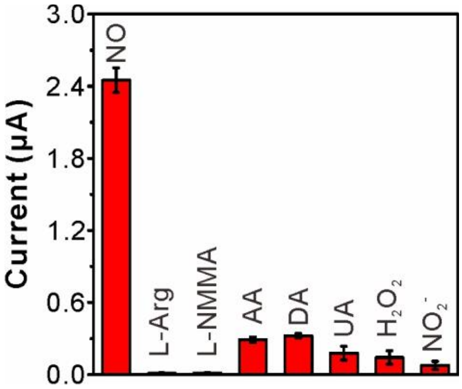

Figure S7. Selective profile of the GRGD/Au NTs/PDMS electrode to interferences. The concentration of each component was 500 nM. The calculated selectivity ratio for NO against ascorbic acid (AA), dopamine (DA), uric acid (UA), H_2O_2 and NO_2^- were 8.4, 7.6, 13.7, 17.3 and 31.7 respectively. The data were presented as mean ± SEM (n = 3, independent experiments).

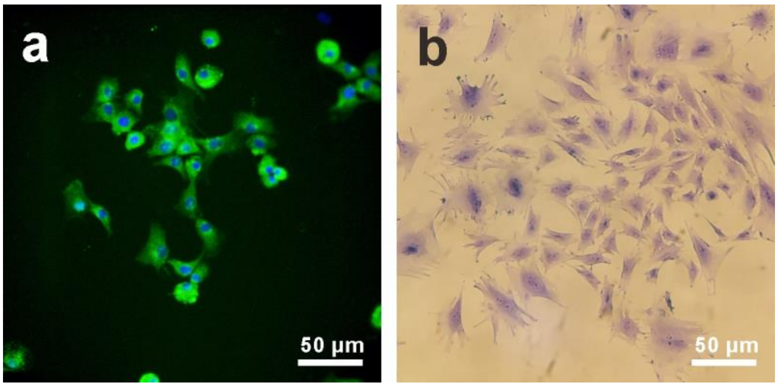

Figure S8. Identification of chondrocytes. (a) Collagen II immunostaining (green: collagen II; blue: nuclei). (b) Toluidine blue staining for proteoglycans.

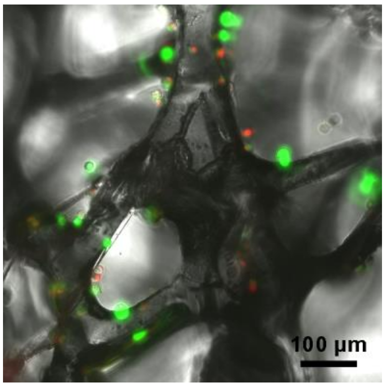

Figure S9. Merged (bright field and fluorescent) image of chondrocytes cultured on Au NTs/PDMS scaffold for 24h and labeled with Calcein-AM (green) and PI (red).

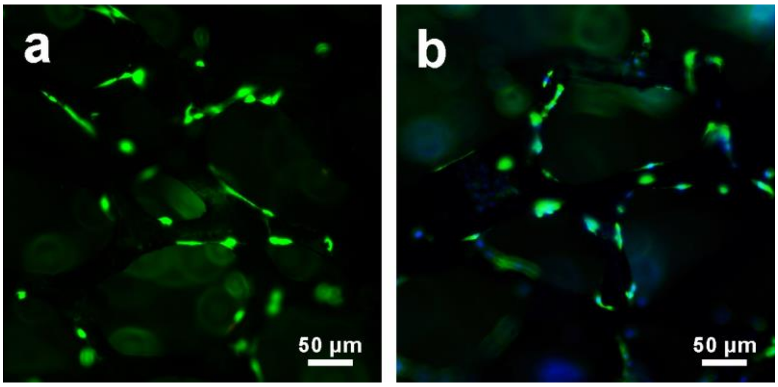

Figure S10. Fluorescent images of chondrocytes cultured on GRGD/Au NTs/PDMS scaffold with an applied voltage (+0.8 V) for 400 s and labeled with (a) Calcein-AM (green)/PI (red) and (b) collagen II (green)/nuclei (blue).

References

[1] a) M. Lashkor, F. J. Rawson, A. Stephenson-Brown, J. A. Preece, P. M. Mendes, *Chem. Commun.* **2014**, *50*, 15589; b) E. Ito, K. Konno, J. Noh, K. Kanai, Y. Ouchi, K. Seki, M. Hara, *Appl. Surf. Sci.* **2005**, *244*, 584.

[2] H. S. Seo, Y. M. Ko, J. W. Shim, Y. K. Lim, J. K. Kook, D. L. Cho, B. H. Kim, *Appl. Surf. Sci.* **2010**, *257*, 596.

[3] A. G. Karakecili, T. T. Demirtas, C. Satriano, M. Gümüsderelioglu, G. Marletta, *J. Biosci. Bioeng.* **2007**, *104*, 69.
